# Supported Molybdenum Carbide and Nitride Catalysts for Carbon Dioxide Hydrogenation

**DOI:** 10.3389/fchem.2020.00452

**Published:** 2020-06-09

**Authors:** Marwa Abou Hamdan, Abdallah Nassereddine, Ruben Checa, Mohamad Jahjah, Catherine Pinel, Laurent Piccolo, Noémie Perret

**Affiliations:** ^1^Univ Lyon, Université Claude Bernard Lyon 1, CNRS, IRCELYON, Villeurbanne, France; ^2^LCIO, Laboratoire de Chimie de Coordination Inorganique et Organométallique, Université Libanaise- Faculté des Sciences I, Beyrouth, Lebanon

**Keywords:** CO_2_ hydrogenation, carbide, nitride, supported catalysts, TiO_2_, ZrO_2_

## Abstract

Catalysts based on molybdenum carbide or nitride nanoparticles (2–5 nm) supported on titania were prepared by wet impregnation followed by a thermal treatment under alkane (methane or ethane)/hydrogen or nitrogen/hydrogen mixture, respectively. The samples were characterized by elemental analysis, volumetric adsorption of nitrogen, X-ray diffraction, and aberration-corrected transmission electron microscopy. They were evaluated for the hydrogenation of CO_2_ in the 2–3 MPa and 200–300°C ranges using a gas-phase flow fixed bed reactor. CO, methane, methanol, and ethane (in fraction-decreasing order) were formed on carbides, whereas CO, methanol, and methane were formed on nitrides. The carbide and nitride phase stoichiometries were tuned by varying the preparation conditions, leading to C/Mo and N/Mo atomic ratios of 0.2–1.8 and 0.5–0.7, respectively. The carbide activity increased for lower carburizing alkane concentration and temperature, i.e., lower C/Mo ratio. Enhanced carbide performances were obtained with pure anatase titania support as compared to P25 (anatase/rutile) titania or zirconia, with a methanol selectivity up to 11% at 250°C. The nitride catalysts appeared less active but reached a methanol selectivity of 16% at 250°C.

## Introduction

The use of carbon dioxide (CO_2_) in current industrial processes is limited to synthesis of urea and its derivatives, carbonates, and salicylic acid, and in the production of methanol from natural gas or coal (Mikkelsen et al., 2010; Wang et al., 2011). With the development of carbon capture technologies, CO_2_ is considered as an attractive renewable resource. By using it as raw material for the synthesis of chemicals, waste CO_2_ might turn into a valuable feedstock (Sakakura et al., 2007). The catalytic hydrogenation of CO_2_ as a C1 building block is one of the promising routes currently investigated (Saeidi et al., 2014).

The catalytic conversion of CO_2_ to CO can occur via reverse water-gas shift (RWGS) reaction. CO is a valuable precursor molecule that can be used for methanol synthesis and for the production of longer-chain hydrocarbons via the Fischer-Tropsch process (Khodakov et al., 2007; Kattel et al., 2017). Today, most of the methanol is synthesized from syngas, i.e., through CO hydrogenation (Olah et al., 2009). An alternative feedstock is CO_2_, where methanol can be produced either via CO_2_ conversion to CO (RWGS) and subsequent hydrogenation, or by the direct hydrogenation of CO_2_ (Wang et al., 2011; Saeidi et al., 2014). Methanol is used in chemical industries, for the synthesis of formaldehyde and acetic acid, the production of dimethyl ether (used as a fuel), or as a solvent (Olah et al., 2009; Alvarado, 2016). Alternatively, the hydrogenation of CO_2_ to methane is also an important catalytic process for CO_2_ valorization (Wei and Jinlong, 2011; Stangeland et al., 2017). Indeed, the methanation (Sabatier's) reaction has gained increasing attention through the “power-to-gas” approach used for energy storage. Throughout this concept, renewable energies such as wind and solar radiation are used to power water electrolysis, generating H_2_. Subsequently, H_2_ is combined with CO_2_ to produce CH_4_. Methane can be stored in classical natural gas infrastructures and be used on demand (Schiebahn et al., 2015).

Tailoring the selectivity to these products depends on both the reaction conditions and the catalyst nature. For example, higher reaction temperatures can lower the selectivity toward methanol that is produced via exothermic reaction, while favoring the selectivity to CO which is produced via the endothermic RWGS reaction (Saeidi et al., 2014). Copper-based catalysts, such as Cu-Ni/Al_2_O_3_, exhibit high selectivity to CO (Liu and Liu, 1999; Wang et al., 2011), and nickel catalysts supported on oxides are the most widely studied materials for methanation (Wei and Jinlong, 2011). Cu/ZnO/Al_2_O_3_ is currently the commercial catalyst for methanol production from H_2_/CO/CO_2_ (Ott et al., 2012). However, these processes still face challenges; for example, one of the major problems with Ni-based catalysts is their deactivation due to carbon deposition and sintering of nickel (Wei and Jinlong, 2011). Furthermore, in methanol synthesis, relatively harsh reaction conditions (220–300°C and 50–100 bar) are used, leading to thermal degradation and sintering of active metal as well as extensive consumption of H_2_ (Ott et al., 2012). Besides Cu or Ni, noble metal-based catalysts are reported to be active and stable in CO_2_ hydrogenation (Kattel et al., 2017). Nevertheless, their high price and scarcity hinder their use.

Recently, transition metal carbides have attracted attention as promising catalysts for the conversion of CO_2_ into CO, methanol, methane, and other hydrocarbons (Nagai et al., 1998b; Solymosi et al., 2002; Porosoff et al., 2014; Posada-Pérez et al., 2014, 2016a,b; Xu et al., 2014; Chen et al., 2015, 2016; Gao et al., 2016; Liu et al., 2016; Han et al., 2019; Reddy et al., 2019). It has been reported through computational work that transition metal (e.g., Ti, Mo, and V) carbide surfaces are able to uptake and activate CO_2_ (Posada-Pérez et al., 2014, 2015; Kunkel et al., 2016, 2018, 2019; Liu et al., 2017). In liquid phase, metals (Pd, Cu, Co, Fe) supported on Mo_2_C have been tested for the hydrogenation of CO_2_ in dioxane under 40 bars, at 135–200°C (Chen et al., 2015, 2016). Pd and Cu promote the formation of methanol while Co and Fe favor ethanol and C_2+_ hydrocarbons (e.g., C_2_H_4_, C_2_H_6_). The hydrogenation of CO_2_ in gas phase has been evaluated over unsupported cubic MoC [particles Xu et al., 2014 or nanowires Gao et al., 2016], hexagonal Mo_2_C [particles Liu et al., 2017; Reddy et al., 2019 or nanowires Gao et al., 2016], Mo_2_C coated with nitrogen and sulfur-codoped carbon (Han et al., 2019), and metals (Au, Cu, Co, Ni) supported on molybdenum carbide (Xu et al., 2015; Posada-Pérez et al., 2016b) or titanium carbide (Rodriguez et al., 2013). The addition of a metal increases the activity and affects the selectivity; e.g., Ni, Co, and Cu favor the formation of methane, hydrocarbons, and methanol, respectively (Xu et al., 2015).

Supported molybdenum carbides have been rarely evaluated for the hydrogenation of CO_2_. At atmospheric pressure and 300°C, Mo_2_C/Al_2_O_3_ (Nagai et al., 1998b) promotes the RWGS reaction. At 220°C and 30 bar, Mo_2_C/MCM-41 exhibits high selectivity to CH_4_ (70–95%), along with CO (0–20%) and methanol (2–27%) at 4 to 10% conversion (Liu et al., 2016). Increasing the Mo_2_C loading from 10 to 40% over MCM-41 increases the selectivity to CO at the expense of CH_4_ and methanol. At 300°C, Mo_x_C_y_/SiO_2_ generates exclusively CO, CH_4_, and H_2_O at atmospheric pressure, while methanol and hydrocarbons (C_2_H_6_, C_3_H_8_) are also obtained at high pressure (20 bars) (García Blanco et al., 2019).

Molybdenum nitride catalysts have been successfully used to promote ammonia synthesis (Hargreaves, 2014), hydrodesulfurization [e.g., thiophene Gong et al., 2005], hydrodenitrogenation [e.g., carbazole Nagai et al., 1998a], and hydrogenation [e.g., p-chloronitrobenzene Perret et al., 2012] reactions. Although they can exhibit similar activity as carbides (Alexander and Hargreaves, 2010), to date only one study reports the use of a nitride for the hydrogenation of CO_2_ (Yao et al., 2019). γ-Mo_2_N is active for the production of CO with selectivity above 98%. The addition of Co increases the conversion in the RWGS reaction.

The synthesis parameters, such as gas composition, gas space velocity, heating rate, and final temperature, influence the final properties of molybdenum nitrides and carbides (Oyama, 1992; Nagai et al., 1998a; Xiao et al., 2000; Hanif et al., 2002; Mo et al., 2016; Cárdenas-Lizana et al., 2018). The presence of a support and its nature also affect the structural and catalytic properties (García Blanco et al., 2019). The crystallographic structure, the metal-to-carbon and metal-to-nitrogen ratio, and the Mo oxidation state are key parameters to be taken in consideration when evaluating the catalytic performances of Mo carbide and nitride catalysts for CO_2_ hydrogenation (Xu et al., 2014; Posada-Pérez et al., 2016a; García Blanco et al., 2019; Han et al., 2019). For example, at 250°C and 20 bar, hexagonal Mo_2_C exhibits higher activity and selectivity for methanation, whereas cubic MoC_1−x_ is less active but more selective to CO and methanol (Xu et al., 2014). However, the opposite trend was observed elsewhere (García Blanco et al., 2019). This might be due to the different synthesis method employed in both studies, i.e., reduction–carburization (Xu et al., 2014) vs. carbothermal reduction route with sucrose (García Blanco et al., 2019). At high temperature [400°C Liu et al., 2017 or 600°C Gao et al., 2016], RWGS occurs and a high selectivity to CO (>98%) is obtained over bulk cubic and hexagonal molybdenum carbides. For Mo_2_C coated with nitrogen and sulfur-codoped carbon, Mo^4+^ favors the synthesis of methanol while methane is formed in the presence of materials with the highest contents of Mo^2+^ and Mo^3+^ (Han et al., 2019).

## Materials and Methods

### Preparation of Catalysts

Commercial TiO_2_-P (TiO_2_ P25, Degussa-Evonik, surface area 55 m^2^ g^−1^), TiO_2_-D (TiO_2_ DT51, Tronox, surface area 85 m^2^ g^−1^), and ZrO_2_ (MEL Chemicals, surface area 129 m^2^ g^−1^) were used as supports. Supported molybdenum carbide and nitride catalysts were prepared by impregnation followed by temperature-programmed reduction–carburization and reduction–nitridation, respectively. First, an appropriate amount (10–12% w/w Mo/support) of ammonium molybdate tetrahydrate (NH_4_)_6_Mo_7_O_24_.4H_2_O (Aldrich Chemical Co, 99.98% trace metals basis) was mixed with 5 g of support and 60 ml of water and stirred for 2 h at room temperature (RT). Water was evaporated under vacuum and the solid was dried in an oven in N_2_ at 80°C overnight. For the second step, the fine powder (1–5 g) was placed in a quartz cell under gas stream and submitted to a thermal treatment.

A range of supported molybdenum carbides was prepared by reduction–carburization under 60 ml min^−1^ (gas hourly space velocity, GHSV = 1090 or 1,530 h^−1^) of *x* % v/v C_2_H_6_/H_2_ or CH_4_/H_2_, with *x* = 5, 10, or 20%. The temperature was increased at 0.5°C min^−1^ up to the final temperature (600, 700, or 800°C) held for 2 h, and then decreased to RT while flowing argon (60 ml min^−1^).

Two different methods were used for the synthesis of supported molybdenum nitride. For method A, a gas stream of 150 ml min^−1^ (GHSV = 8,200 h^−1^) of 17% v/v N_2_/H_2_ was used. The temperature was raised at 5°C min^−1^ to 700°C and held for 2 h, and then decreased to RT while flowing N_2_ (60 ml min^−1^). For method B, a gas stream of 432 ml min^−1^ (GHSV = 23,600 h^−1^) of 83% v/v N_2_/H_2_ was used. The temperature was raised at 9.2°C min^−1^ to 300°C, then at 0.6°C min^−1^ to 500°C, then at 2°C min^−1^ to 700°C, held for 2 h, and then decreased to RT while flowing N_2_ (60 ml min^−1^).

The non-passivated (NP) samples were transferred into the reactor via a glovebox, in order to avoid exposure to air. All the other samples were passivated for 4 h under 1% v/v O_2_/N_2_ (60 ml min^−1^).

MoO_3_/TiO_2_**-**D was prepared by impregnation followed by calcination under 180 ml min^−1^ of air. The temperature was increased at 0.5°C min^−1^ to 600°C, held for 2 h, and cooled down to RT.

### Characterization of Catalysts

The Mo content of the catalysts was determined by inductively coupled plasma optical emission spectroscopy (ICP-OES) with an Activa instrument from Horiba Jobin Yvon. Before analysis, the samples were mineralized by fusion with lithium tetraborate in Pt-Au crucibles at 1,100°C and then soaked with 20% HCl.

Carbon elemental analyses were conducted via a Leco SC144 micro-analyzer. The total combustion of the samples was performed at 1,050°C under a stream of helium and oxygen. The carbon was converted to carbon dioxide and quantified by a thermal conductivity detector (TCD).

Nitrogen elemental analyses were performed with a FLASH 2000 analyzer from ThermoFisher. The oxidation/reduction reaction of nitrogen-containing species into N_2_ gas was performed at 950°C with an O_2_ flow of 90 ml/min for 17 s. N_2_ was analyzed via separation on a packed column and detection with a TCD. Helium was used as a carrier gas with a flow of 140 ml/min and as a reference for the detector. A three-point calibration curve was built for quantification with methionine calibration samples purchased from ThermoFisher.

BET surface areas of the samples were determined from N_2_ physisorption after desorption at 150°C for 3 h under ultra-high vacuum (10^−4^ mbar) using an ASAP 2020 Micromeritics apparatus. The values are reported with an absolute precision of ± 5 m^2^ g^−1^.

Powder X-ray diffraction (XRD) patterns of the catalysts were recorded in the range 2θ = 10–90° at 0.04° s^−1^ using a Bruker D8 A25 X-ray diffractometer and a CuK_α_ radiation source (λ = 1.54184 Å). Phase identification, lattice parameters, and mean crystallite sizes (*d* = 4/3 × *LVol-IB*, with *LVol-IB* being the volume-averaged column height) were obtained by performing Rietveld refinement using Topas 5 software.

Transmission electron microscopy (TEM) and scanning TEM–high angle annular dark field (STEM-HAADF) images were obtained using an image-aberration-corrected FEI Titan ETEM G2 instrument operated at 300 kV, equipped with an X-MAX SDD EDX detector from Oxford Instruments. Samples were prepared by dispersing the solids in ethanol and then depositing them onto carbon-coated copper grids.

### Catalytic Testing

The catalyst evaluation in CO_2_ hydrogenation was carried out in a fixed-bed flow reactor. In standard conditions, a mass of 400 mg of the catalyst was placed in a tubular Pyrex glass reactor comprising a glass frit. This borosilicate tube was inserted into a stainless-steel U-shaped cover. A graphited Teflon gasket was put around the top of the borosilicate tube to hug the metal cover and pushed by a metal gasket, ensuring at the same time full flow inside the tube reactor and sealing of the cover. The reactor set was placed in a tubular resistive oven. The system was purged with the H_2_/CO_2_/N_2_ (30/10/10 ml/min) reaction mixture and then kept exposed to this flow till reaching a total pressure of 30 bar controlled with a back-pressure regulator, at which heating to 250°C was started. Every valve and connection was heated to maintain the mixture in gas phase for online analyses. All parameters of flow, pressure, and temperature were recorded using a home-made Labview program. The reaction was performed for 3 h. Some of the reaction conditions were varied: the mass of catalyst (400 or 800 mg), the flow of H_2_/CO_2_/N_2_ (30/10/10 or 50/10/10 ml/min), the reaction temperature (from 200 to 300°C), and the pressure (20 or 30 bar).

The reaction system was connected to a R3000 micro gas chromatograph (MicroGC) with a TCD detector, purchased from SRA Instruments, with which samples were taken every 3 min during the reaction. Three modules were used to analyze the gases. H_2_, N_2_, CO, and CH_4_ were analyzed with a 10-m Molsieve 5 A capillary column with a backflush injector and a 3-m PlotU precolumn. An 8-m Poraplot U capillary column was used for CO_2_, C_2_-C_3_ hydrocarbons, methanol, and water. A 10-m Stabilwax capillary column was employed for oxygenated compounds such as dimethyl ether, alcohols, and water as well. The MicroGC was calibrated and operated by using the Soprane software. For the determination of catalytic performances from GC data, we used the following definitions of conversion and selectivity:

(1) $$\begin{array}{l} CO_{2}\;conversion\;=\;\frac{F{\left(CO_{2}\right)}_{in}\;-\;F{\left(CO_{2}\right)}_{out}}{F{\left(CO_{2}\right)}_{in}} \end{array}$$

where *F(CO*_2_*)*_*in*_ and *F(CO*_2_*)*_*out*_ are the incoming and outgoing flow rates of CO_2_, respectively.

(2) $$\begin{array}{l} Selectivity\;to\;product\;P\;=\;\frac{n_{c}\left(P\right)\;F{\left(P\right)}_{out}\;}{F{\left(CO_{2}\right)}_{in}\;-\;F{\left(CO_{2}\right)}_{out}} \end{array}$$

where *n*_*c*_(*P*) is the number of carbon atoms per molecule of P and *F(P)*_*out*_ is the (outgoing) flow rate of product P. Conversion and selectivity are given with an error of 8 and 7%, respectively.

## Results and Discussion

### Preparation and Characterization of Molybdenum Carbide Catalysts

A series of molybdenum carbide catalysts supported on TiO_2_-P, TiO_2_-D, and ZrO_2_ were synthesized from ethane and methane. The preparation conditions are reported in Table 1 and Table S1: molar fraction of hydrocarbon in H_2_ (from 5 to 20%), maximal temperature (600°C−800°C), and GHSV employed during reduction–carburization (1,090 or 1,530 h^−1^).

**Table 1:** List of the supported molybdenum carbide catalysts synthesized from C_2_H_6_/H_2_, with the corresponding preparation conditions, the C/Mo atomic ratio derived from elemental analyses, and the support composition (% phase).

**Entry**	**Catalyst name**	**GHSV (h^**−1**^)**	**Gas stream**	***T*_**max**_ (^**°**^C)**	**C/Mo**	**% phase**
(C1)	MoC/TiO_2_-P	1,530	20% C_2_H_6_/H_2_	700	1.5	75^a^
(C2)	MoC_5E−700_/TiO_2_-P	1,090	5% C_2_H_6_/H_2_	700	0.7	55^a^
(C3)	MoC_10E−700_/TiO_2_-P	1,090	10% C_2_H_6_/H_2_	700	0.7	71^a^
(C4)	MoC_20E−700_/TiO_2_-P	1,090	20% C_2_H_6_/H_2_	700	1.0	74^a^
(C5)	MoC_20E−600_/TiO_2_-P	1,090	20% C_2_H_6_/H_2_	600	0.7	75^a^
(C6)	MoC_20E−800_/TiO_2_-P	1,090	20% C_2_H_6_/H_2_	800	1.3	n.a.
(C7)	MoC_20E−700_/TiO_2_-D	1,090	20% C_2_H_6_/H_2_	700	1.4	100^a^
(C8)	MoC_20E−700_/ZrO_2_	1,090	20% C_2_H_6_/H_2_	700	1.8	88^b^

*The name of the samples (MoC) does not reflect their C/Mo ratio*.

a *Anatase/rutile composition (% anatase)*.

b *Monoclinic/tetragonal composition (% monoclinic)*.

The MoC/TiO_2_**-**P catalyst (entry C1, Table 1) was first synthesized and used to test several reaction conditions for the hydrogenation of CO_2_. However, a high GHSV (1,530 h^−1^) was employed during the synthesis, which resulted in a high carbon content (2.2 wt%) and an excess of carbon on the surface. Our group previously reported that the carbon content decreases as the GHSV decreases (Abou Hamdan et al., 2019), which can be ascribed to the higher hydrocarbon decomposition rate at higher flow rate (i.e., GHSV) (Solymosi et al., 1994). Therefore, the other catalysts were synthesized with a lower GHSV (1090 h^−1^) in order to limit the formation of graphite.

The molybdenum (9.1–11.8 wt%) and carbon (0.3–2.2 wt%) contents are reported in Table S2. The C/Mo atomic ratios (Table 1 and Table S1) are between 0.2 and 1.5, knowing that this value should be between 0.5 (Mo_2_C) and 1 (MoC) for stoichiometric fully carburized molybdenum carbide. As expected, MoC_20E−700_/TiO_2_**-**P (C4, Table 1) exhibits a lower C/Mo ratio than MoC/TiO_2_**-**P (1.0 vs. 1.5) as the GHSV is lower and the other parameters being identical. MoC_20M−600_/TiO_2_-P and MoC_5M−700_/TiO_2_**-**P are only partially carburized, owing probably to the low temperature and the low amount of methane in the gas stream, respectively, during reduction–carburization. It is expected that methane decomposition increases with increasing methane concentration in the methane/hydrogen mixture, as reported elsewhere (Ostrovski and Zhang, 2006). Consequently, the C/Mo ratio increases as the hydrocarbon concentration increases and reaches unity for MoC_20E−700_/TiO_2_**-**P. In addition, the degree of carburization is higher when using ethane instead of methane, as the carburization occurs at lower temperature (Hanif et al., 2002). A C/Mo ratio superior to 1.0 indicates an excess of carbon, associated with the presence of amorphous graphite on the surface, as demonstrated elsewhere by Raman analyses (Abou Hamdan et al., 2019). This is the case for the samples synthesized with a high amount of ethane, a high temperature or a high GHSV. This phenomenon is also more pronounced for TiO_2_-D and ZrO_2_ than for TiO_2_-P, suggesting an influence of the support on the decomposition of the hydrocarbon. There is evidence in the literature on supported Ni and Pd systems that CH_4_ decomposition depends on metal crystallite size, metal-support interaction, nature, and crystal phase of the support (e.g., tetragonal vs. monoclinic ZrO_2_) and on acid–base properties of the materials (Solymosi et al., 1994; Bengaard et al., 2002; Zhang et al., 2019). However, we were unable to find any related study for supported Mo or MoO_x_ systems. It is worth pointing out that hydrocarbon decomposition and reduction of the precursor occur at the same time, which complicates the analysis. However, it is beyond the scope of this paper to go into a detailed discussion of hydrocarbon decomposition.

**Figure 1:**
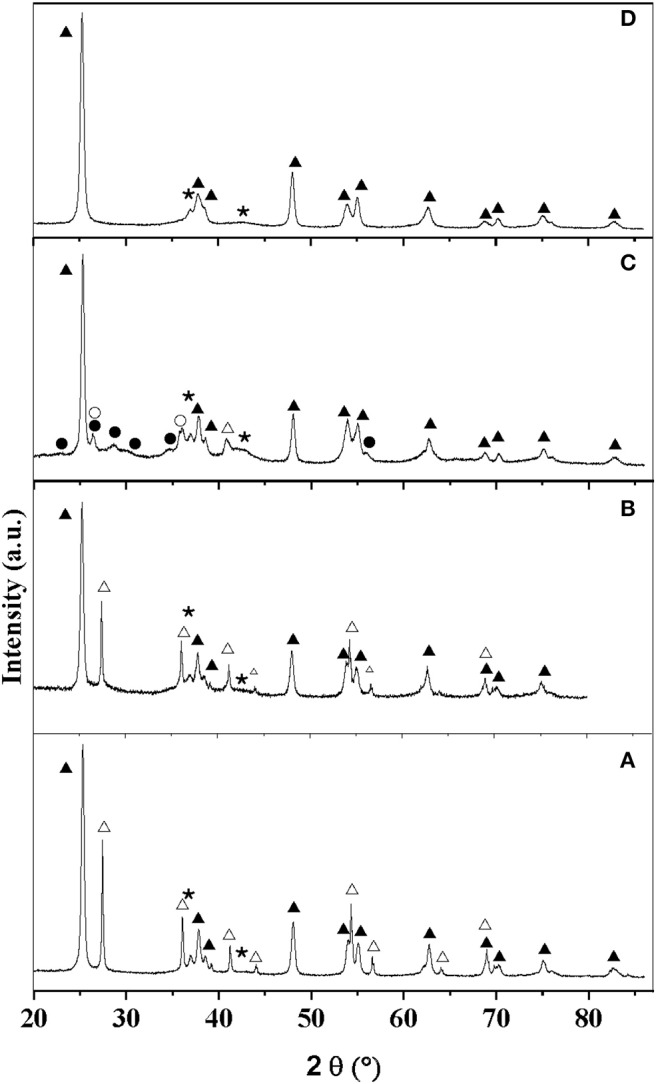
XRD patterns of the catalysts: **(A)** MoC_10E−700_/TiO_2_**-**P, **(B)** MoC/TiO_2_**-**P, **(C)** MoC_20E−800_/TiO_2_**-**P, and **(D)** MoC_20E−700_/TiO_2_-D. Assignment of peaks: (▴) anatase, (△) rutile, (•) Ti_5_O_9_, (○) Ti_4_O_7_, and (⋆) MoC.

All the samples were characterized by XRD. The patterns associated with MoC_10E−700_/TiO_2_**-**P, MoC/TiO_2_**-**P, MoC_20E−800_/TiO_2_**-**P, and MoC_20E−700_/TiO_2_-D are presented in Figure 1, while all the others are included in Figures S1, S2. The patterns obtained for the catalysts synthesized at 600°C and 700°C present mainly the peaks associated with the support: a mixture of anatase and rutile phases for TiO_2_-P, anatase for TiO_2_-D, and a mixture of monoclinic and tetragonal phases for ZrO_2_. With catalysts based on TiO_2_-P (75% anatase/25% rutile), the final amount of anatase is between 55 and 75%, depending on the synthesis conditions (Table 1 and Table S1). In presence of Mo carbide, the anatase is stable up to 700°C under 20% hydrocarbon/H_2_ while it transforms into rutile under 5% and 10% hydrocarbon/H_2_ gas streams. Thus, the presence of fully carburized Mo carbide and/or free carbon seems to stabilize the anatase phase. However, after reduction–carburization at 800°C, the support is partially reduced to Ti_5_O_9_ and Ti_4_O_7_ (Figure 1C and Figure S2F). Finally, the crystallite sizes of the anatase phase (26–29 nm) are similar while the ones associated with rutile vary from 11 to 82 nm (Table S2). Regarding the catalysts based on TiO_2_-D, the anatase phase is stable, i.e., no rutile was observed and the crystallite size is similar to the bare support (ca. 26 nm). This agrees with the results observed for TiO_2_-P at 700°C under 20% hydrocarbon/H_2_. In the same manner, the ZrO_2_ support is stable, with a composition that remains at 88% monoclinic/12% tetragonal.

The specific surface area of Mo carbide supported on TiO_2_-P and synthesized at 600°C (54 m^2^ g^−1^; Table S2) is similar to that of the bare support (55 m^2^ g^−1^). However, these values decrease to 43 m^2^ g^−1^ as the percentage of anatase decreases to 55%. Finally, the surface area is of 28 m^2^ g^−1^ due to the reduction of the support after treatment at 800°C, for MoC_20M−800_/TiO_2_**-**P. The catalysts supported on TiO_2_-D and ZrO_2_ exhibit surface areas similar to those of the bare supports (129 m^2^ g^−1^ for ZrO_2_; 90 m^2^ g^−1^ for TiO_2_-D).

XRD analyses suggest the formation of fcc MoC in all the samples. This phase is characterized by two main peaks at 36.4° and 42.2°, associated with the (111) and (002) planes (PDF 01-089-2868). While the first one overlaps with the peaks associated with the supports, the second one can be more easily observed. The Mo carbide crystallite sizes were estimated by Rietveld refinement to be around 2–4 nm for all the samples (Table S2).

**Figure 2:**
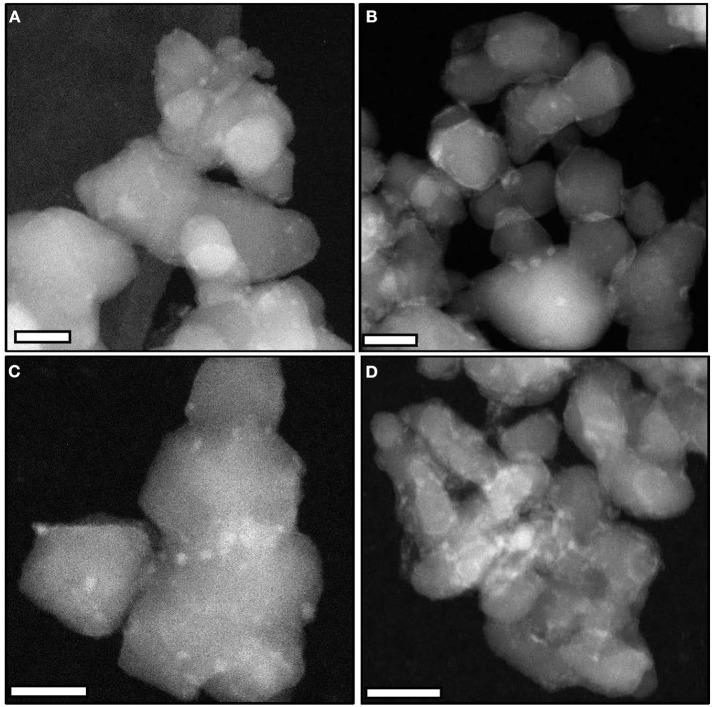
Representative STEM-HAADF images (scale bars, 20 nm) of **(A)** MoC/TiO_2_**-**P, **(B)** MoC_10E−700_/TiO_2_**-**P, **(C)** MoC_10M−700_/TiO_2_**-**P, and **(D)** MoC_20E−700_/TiO_2_-D.

STEM-HAADF images of the catalysts (Figure 2) confirm the presence of small particles (size < 4 nm) at the surface of the supports, independently of the synthesis conditions. The interplanar distances and angles estimated by electron diffraction and TEM analyses on selected samples (MoC_20E−700_/TiO_2_-D: Figure S3 and Table S3; MoC_10E−700_/TiO_2_**-**P: Figure S4 and Table S4) confirm the formation of the MoC phase with fcc structure.

### Preparation and Characterization of Molybdenum Nitride Catalysts

In order to compare the nature of the atoms incorporated into the structure, a series of supported molybdenum nitride catalysts were synthesized using method A (MoN_A_) or method B (MoN_B_) with TiO_2_-P, TiO_2_-D, or ZrO_2_ as support (Table 2). Based on the literature dealing with the synthesis of bulk molybdenum nitride (Ghampson et al., 2012; Perret et al., 2012; Cárdenas-Lizana et al., 2018), the formation of hexagonal β-Mo_2_N is favored when using low GHSV, high heating ramp and low N_2_ content in the gas stream (method A) while the opposite conditions usually generate cubic γ-Mo_2_N with high surface area (method B).

**Table 2:** List of the supported molybdenum nitride catalysts with the corresponding preparation conditions, the N/Mo atomic ratio derived from elemental analyses, and the support composition (% phase).

**Entry**	**Catalyst**	**Gas stream**	**GHSV (h^**−1**^)**	**N/Mo**	**% phase**
(N1)	MoN_A_/TiO_2_**-**P	16%N_2_/H_2_	8,200	0.48	65^a^
(N2)	MoN_B_/TiO_2_**-**P	84%N_2_/H_2_	27,600	0.57	69^a^
(N3)	MoN_A_/TiO_2_-D	16%N_2_/H_2_	8,200	0.55	100^a^
(N4)	MoN_B_/TiO_2_-D	84%N_2_/H_2_	27,600	0.70	100^a^
(N5)	MoN_A_/ZrO_2_	16%N_2_/H_2_	8,200	0.47	85^b^
(N6)	MoN_B_/ZrO_2_	84%N_2_/H_2_	27,600	0.51	86^b^

*The name of the samples (MoN) does not reflect their N/Mo ratio*.

a *Anatase/rutile composition (% anatase)*.

b *Monoclinic/tetragonal composition (% monoclinic)*.

The molybdenum loading is around 9% for all the catalysts (Table S5). The N/Mo ratios presented in Table 2 are around 0.5, suggesting the formation of Mo_2_N. For all the samples, the ratios were slightly higher when synthesized with method B as compared to method A. MoN_B_/TiO_2_-D exhibits a ratio of 0.70, slightly above what has been reported in the literature for bulk molybdenum (N/Mo ratios: 0.3–0.5) (Gong et al., 2006; Kong et al., 2008; Cairns et al., 2009; Perret et al., 2012). This might be due to the incorporation of some nitrogen atoms in interstitial sites and defects of TiO_2_. This has previously been reported for molybdenum nitride supported on Al_2_O_3_ and SBA-15 (Ghampson et al., 2012; Zheng et al., 2013).

**Figure 3:**
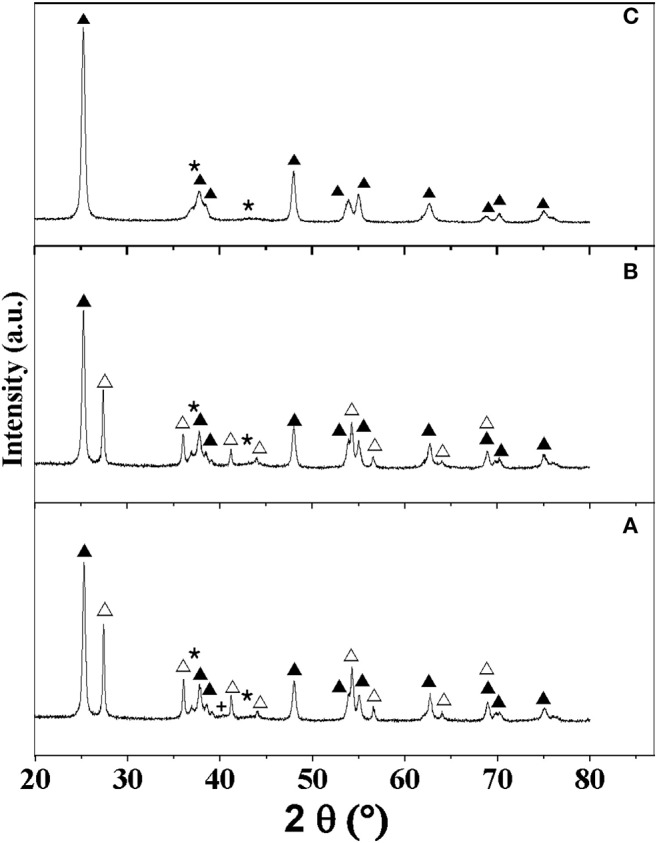
XRD patterns of the catalysts: **(A)** MoN_A_/TiO_2_**-**P, **(B)** MoN_B_/TiO_2_**-**P, and **(C)** MoN_B_/TiO_2_-D. Assignment of peaks: (▴) anatase, (△) rutile, (⋆) MoN, and (**+**) Mo.

The diffraction patterns associated with MoN_A_/TiO_2_**-**P, MoN_B_/TiO_2_**-**P, and MoN_B_/TiO_2_-D are presented in Figure 3, while the other ones are reported in Figure S5. Rietveld refinements suggest the presence of small crystallites of cubic Mo nitride in all the samples (Table S5). However, the two main peaks of the cubic and hexagonal phases of Mo_2_N are close to each other (37.3°/43.3° and 37.3°/43.1°, respectively). As the minor diffraction lines were not observable by XRD due to the low loading and the small size, it is difficult to discriminate between the two phases. For MoN_A_/TiO_2_**-**P and MoN_A_/TiO_2_-D, an additional peak at 40° with a small intensity can be attributed to metallic Mo. In agreement with the elemental analyses, the results suggest that method A does not allow a full nitridation of the samples; hence, some metallic Mo is still present. Similarly to supported carbides, the thermal treatment under reducing atmosphere is associated with a slight transformation of anatase to rutile (from 75 to 65–69% of anatase) for TiO_2_-P, while TiO_2_-D and ZrO_2_ remain stable (Table 2).

**Figure 4:**
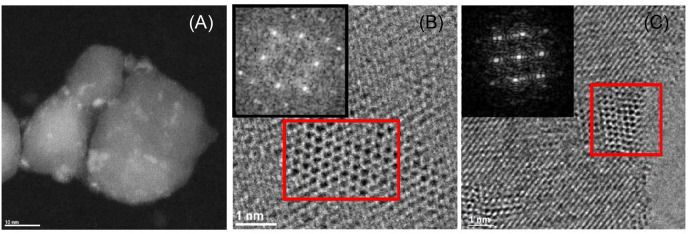
Representative STEM image of MoN_B_/TiO_2_-D **(A)**; TEM images and electron diffraction patterns (corresponding to the red squares in the images) of MoN_B_/TiO_2_-D **(B)** and MoN_A_/ZrO_2_ **(C)**.

The catalysts were also characterized by STEM, TEM, and electron diffraction, as shown in Figure 4. The catalysts exhibit small (<5 nm) Mo nitride nanoparticles. Moreover, the d-spacing and angles estimated for some particles correspond to cubic Mo_2_N (Figures S6, S7 and Tables S6, S7), in agreement with XRD.

### CO_2_ Hydrogenation Over Supported Molybdenum Carbide

#### Preliminary Catalytic Test Over MoC/TiO_2_-P

**Figure 5:**
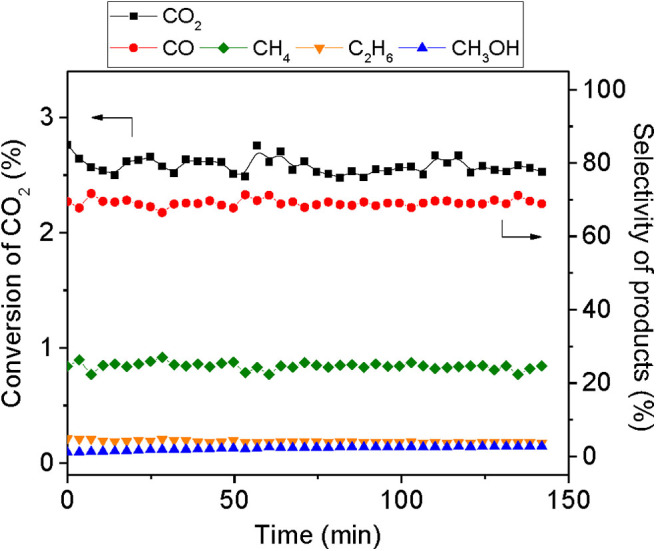
Temporal evolution of CO_2_ conversion and selectivity of products during the hydrogenation of CO_2_ over 400 mg of MoC/TiO_2_-P at 250°C and 20 bar total pressure, with a total flow rate of 30 ml min^−1^ of H_2_ /CO_2_/N_2_ with a H_2_:CO_2_ ratio of 5:1, 150 min on stream after stabilization of the temperature.

All the preliminary tests were conducted with MoC/TiO_2_**-**P (C1, Table 1). For the first evaluation, the reaction conditions used by Rodriguez and coworkers (Xu et al., 2014) were employed. The reaction was performed at 250°C, with a total pressure of 20 bar and a total flow rate of 30 ml min^−1^ using a reaction mixture of 75% H_2_/15% CO_2_/10% N_2_. Figure S8A shows the temporal evolution of CO_2_ conversion and product yields. CO_2_ conversion started around 190°C; it increased with the temperature and reached a maximum (3.5%) at 113 min corresponding to the overshooting of the temperature of the oven (260°C). The evolution of the product selectivity as a function of temperature, shown in Figure S8B, reveals that, at low temperature, only CO was produced. Afterwards, a decrease in CO selectivity was observed, associated with a corresponding increase in CH_4_. In parallel, C_2_H_6_ and CH_3_OH were formed in small quantities; however, the selectivity of the former product gradually increased. Figure 5 presents CO_2_ conversion (around 2.5%) and selectivity to products once the temperature is stabilized at 250°C. At quasi-steady state, the major product is CO with a selectivity of 69%, followed by CH_4_ (24%), C_2_H_6_ (4%), and CH_3_OH (3%).

These results suggest that MoC/TiO_2_**-**P behaves primarily as a RWGS catalyst. According to the molybdenum carbide content, the conversion (2.5% for 56 mg of MoC) is similar to the one reported for a cubic molybdenum carbide catalyst under the same reactions conditions (11% conversion over 200 mg of MoC) (Xu et al., 2014). However, the product distribution is different and the present catalyst shows higher selectivity to CO and CH_4_ with lower selectivity to methanol. Indeed, the authors reported that cubic MoC is selective to CO (51%), CH_3_OH (23%), CH_4_ (16%), and C_2_H_6_ (3%), whereas hexagonal MoC leads to less CO (34%) and CH_3_OH (12%), but more CH_4_ (37%) and C_2_H_6_ (9%). The authors proposed that methane and ethane would result from the hydrogenation of the carbon generated from the full decomposition of CO_2_ on the surface. Our results are closer to the ones reported by García Blanco et al. (2019) over cubic MoC_1−x_ under similar conditions (240°C, 20 bar). They measured an 8% conversion, with selectivity to CO (55%), CH_4_ (30%), C_2_H_6_ (4%), and methanol (3%).

#### Screening of the Reaction Conditions Over MoC/TiO_2_-P

Several reaction conditions were assessed by varying the total gas flow rate, the H_2_/CO_2_ molar ratio, the temperature, and the catalyst weight. Table S8 reports the CO_2_ conversion and product selectivity results at quasi-steady state. First, the reproducibility and the absence of mass transfer limitations were checked. Afterwards, the total flow rate of the reactant mixture was decreased from 50 ml min^−1^ to 10 ml min^−1^. As expected, decreasing the flow rate increased the conversion to the same extent (from 4 to 12%). The selectivity to CH_4_ and C_2_H_6_ increased at the detriment of CO and methanol.

The effect of temperature on the reaction kinetics was investigated for 200, 250, and 300°C. When the temperature increases, the quasi-steady-state conversion increases from 0.5 to 11.4%. This is associated with a slight increase in selectivity toward CO (from 72 to 79%) and C_2_H_6_ (from 0 to 3%), along with a decrease in selectivity toward CH_4_ (from 26 to 18%) and methanol (from 2 to 0.4%). Less methanol is expected when the temperature is increased, according to the Le Chatelier principle (Romero-Saez et al., 2019). However, as far as CO and methane are concerned, Xu et al. (2014) measured a lower CO selectivity and a higher CH_4_ selectivity at higher temperature for cubic MoC.

Finally, the influence of the passivation treatment on the catalytic performance of MoC/TiO_2_**-**P was evaluated by conducting another test with a fresh non-passivated catalyst transferred into the reactor through a glovebox. This catalyst appears more active than the passivated one, while the product selectivity is unaltered. This is consistent with the study of Nagai et al. (1998b), who showed that passivation decreases the activity of Mo carbide catalysts supported on alumina in CO_2_ hydrogenation at 300°C and atmospheric pressure (however, the authors did not mention whether passivation had an influence on the selectivity).

#### Effect of the Catalyst Carburization Conditions on the Activity of MoC/TiO_2_-P

MoC/TiO_2_-P catalysts were prepared with different methane or ethane concentrations (5, 10, and 20%) and carburization temperatures (600, 700, and 800). The XRD, STEM, and TEM analyses confirmed the formation of well-dispersed fcc MoC particles on the support. The main difference between these catalysts lies in the C/Mo ratios.

**Figure 6:**
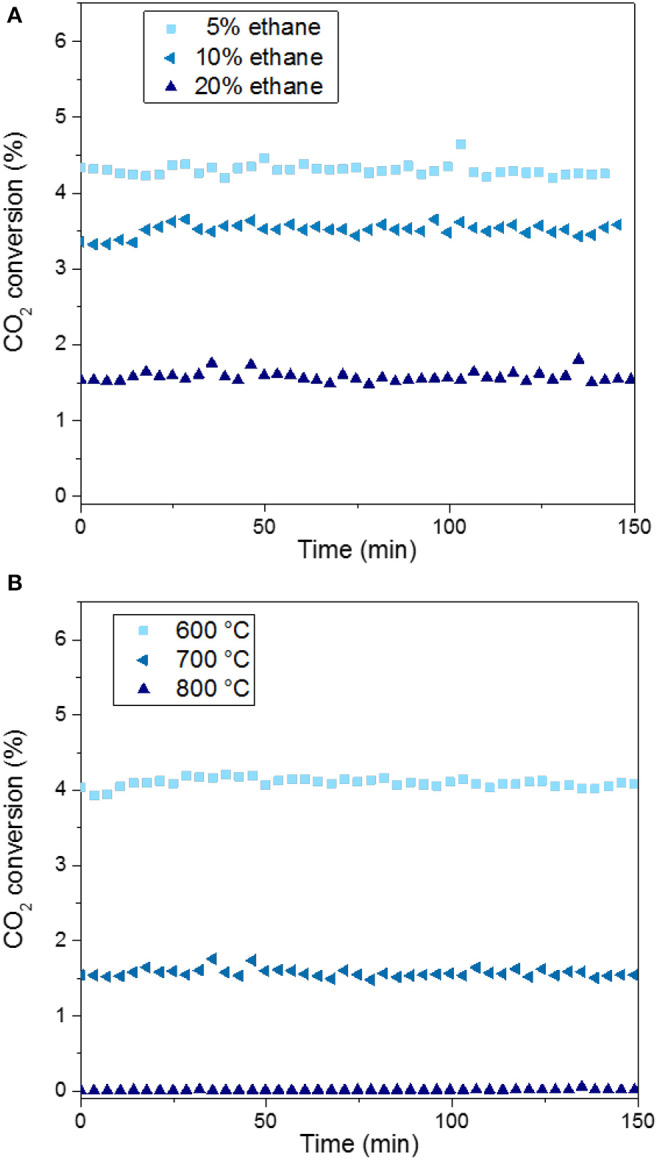
CO_2_ conversion over MoC/TiO_2_-P as a function of the amount of ethane used for carburization at 700°C **(A)** and the carburization temperature used for 20% ethane **(B)**. Catalytic conditions: 400 mg of catalyst, 50 ml min^−1^, 250°C, 30 bar, H_2_:CO_2_ = 3:1, 150 min on stream after stabilization of the temperature.

**Figure 7:**
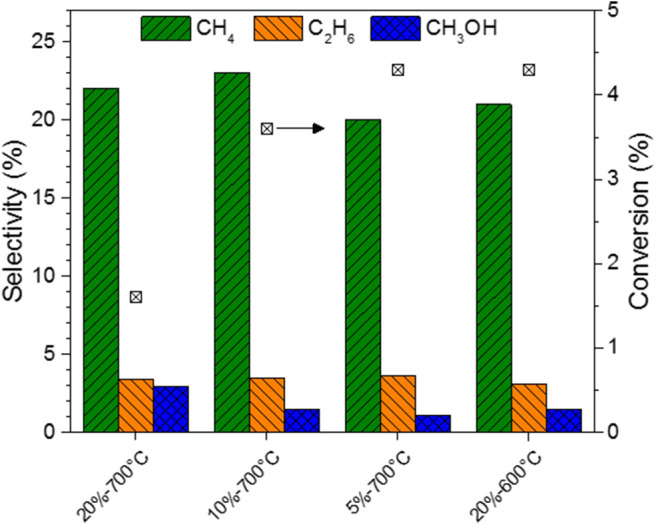
Selectivity to methane, ethane, and methanol, and conversion for MoC/TiO_2_-P as a function of the carburizing ethane concentration (5, 10, or 20% in H_2_), and the carburization temperature (600 or 700°C). Catalytic conditions: 400 mg of catalyst, 50 ml/min, 250°C, 30 bar, H_2_:CO_2_ = 3:1, 150 min on stream after stabilization of the temperature.

The evolution of CO_2_ conversion with time-on-stream for MoC/TiO_2_-P catalysts prepared with ethane is presented in Figure 6. Figure 7 reports the corresponding product distributions at quasi-steady state (CO is not shown). The general trend is that CO_2_ conversion decreases with the increase in carbon content (higher C/Mo ratio, Figure S9), which is in accordance with the literature (Posada-Pérez et al., 2014; Xu et al., 2014). Substantial increases in the selectivity toward CO and methanol were reported when moving from a C-rich surface to a Mo-rich surface. In our case, the selectivity to CO (72–75%), methane (20–23%), and ethane (3%) does not significantly differ between the catalysts. However, differences appear in terms of methanol selectivity, which varies from 1 to 3%. It is worth noting that all the conversions and selectivity remained stable with time. A previous study on supported molybdenum carbide (Mo_2_C/MCM-41) reported that the conversion decreased with time and the selectivity varied (Liu et al., 2016).

Regarding the catalysts synthesized at 700°C (Figure 6A), the higher the alkane concentration during the catalyst preparation, the lower the CO_2_ conversion is. The preparation of the carbide with a low concentration (5%) of ethane favors CO_2_ conversion: 4.3% for MoC_5E−700_/TiO_2_**-**P, which is the highest value. In contrast, a high concentration of ethane (MoC_20E−700_/TiO_2_**-**P) leads to the highest selectivity to methanol (3%), but at the lowest conversion (1.5%).

The performances of the catalysts prepared with ethane at different carburization temperatures are shown in Figure 6B. The conversion decreases from 4% to near-zero value with the increase in the carburization temperature from 600 to 800°C. The graphitic carbon present on the surface of the fully carburized catalysts may block the access of the reactants to the active surface of the material, i.e., it may act as a poison, as previously reported (Nagai et al., 1998b). The support undergoes significant phase changes at 800°C (section Preparation and Characterization of Molybdenum Carbide Catalysts), which might also explain the lack of activity of MoC_20E−800_/TiO_2_**-**P. As this catalyst was barely active, its selectivity is not reported in Figure 7.

The same trends are observed when using methane as carburizing hydrocarbon (Figures S10, S11). Thus, the increase in carbon content of the carbide appears to moderate its activity, consistently with previous work (Posada-Pérez et al., 2016a). The selectivity to methanol increases up to 3% with the increase in temperature of preparation or hydrocarbon content, when using ethane. The highest selectivity to methanol (5.3%) is observed for the catalyst with the lowest C/Mo ratio, i.e., MoC_20M−600_/TiO_2_**-**P.

#### Effect of the Support Nature

The nature of the support is known to affect catalytic performances. For example, Kim et al. have shown that the support crystal structure of Ru/TiO_2_ strongly affects the hydrogenation of CO_2_ (Kim et al., 2018). Moreover, PtCo/ZrO_2_ and PtCo/TiO_2_ exhibit distinct selectivity in CO_2_ hydrogenation, the former catalyst being more selective to CH_4_ (Kattel et al., 2016). In both studies, the differences in behaviors were attributed to distinct metal–support interactions in the catalysts.

The performances of Mo carbide catalysts synthesized from ethane at 700°C and supported on TiO_2_**-**P, TiO_2_**-**D, and ZrO_2_ are compared in Table 3. The catalyst supported on TiO_2_**-**D (pure anatase) shows a higher conversion than those supported on TiO_2_**-**P and ZrO_2_. This is in agreement with the results obtained with Ru/TiO_2_, where the conversion of CO_2_ increases with the anatase content in the support (Kim et al., 2018). A significant difference is observed in the product selectivity, which is affected by the nature of the support more than by the C/Mo ratio. The selectivity to methanol of the catalyst supported on TiO_2_-D is superior by ca. 8% compared to the case of TiO_2_**-**P. This catalyst also shows lower CO and CH_4_ selectivity. The selectivity of MoC_20E−700_/ZrO_2_ is similar to that of MoC_20E−700_/TiO_2_**-**P. The same trends are observed for the catalysts synthesized using methane as hydrocarbon (Table S9). These results make the use of TiO_2_**-**D preferable over the other supports as CO_2_ conversion and methanol selectivity both increase in the order: MoC supported on TiO_2_**-**D > TiO_2_**-**P ≈ ZrO_2_.

**Table 3:** Effect of the support nature on the catalytic performances.

**Catalyst**	**CO_**2**_ conversion (%)**	**Product selectivity (%)^a^**			
		**CO**	**CH_**4**_**	**C_**2**_H_**6**_**	**CH_**3**_OH**
MoC_20E−700_/TiO_2_**-**P	1.6	71	22	4	3
MoC_20E−700_/ TiO_2_-D	2.2	68	16	5	11
MoC_20E−700_/ZrO_2_	1.7	69	24	3	4
MoO_3_/TiO_2_-D	0.5	77	15	0	8

a *Reaction conditions: 400 mg of catalyst, 50 ml/min, 250°C, 30 bar, 3:1 H_2_:CO_2_ ratio, 280 min on stream*.

The active sites reported for molybdenum carbide catalysts in the hydrogenation of CO_2_ are Mo-terminated and C-terminated Mo_2_C (Posada-Pérez et al., 2016b). Molybdenum and carbon defects are also present at the surface and may affect the catalytic response (de Oliveira et al., 2014). As the conversion increases with the decrease in C/Mo ratio (section Effect of the Catalyst Carburization Conditions on the Activity of MoC/TiO_2_-P), our results suggest that the amount of carbon vacancies affects the catalytic performance and/or that the C-terminated sites are inactive. The supported carbides were compared with MoO_3_/TiO_2_**-**D in order to assess the respective roles of molybdenum and carbon species. The XRD diffraction pattern (Figure S12) confirmed the formation of orthorhombic MoO_3_. A very low conversion (0.5%) was measured with this catalyst (Table 3); hence, Mo^6+^ are not active for this reaction. We have previously shown that MoC/TiO_2_ exhibits mainly Mo^δ+^ (δ^+^ < 2) species (Abou Hamdan et al., 2019). Therefore, it seems that the combination of Mo^δ+^ (δ^+^ < 2) species with C vacancies is favorable to the hydrogenation of CO_2_.

### CO_2_ Hydrogenation Over Supported Molybdenum Nitride

**Figure 8:**
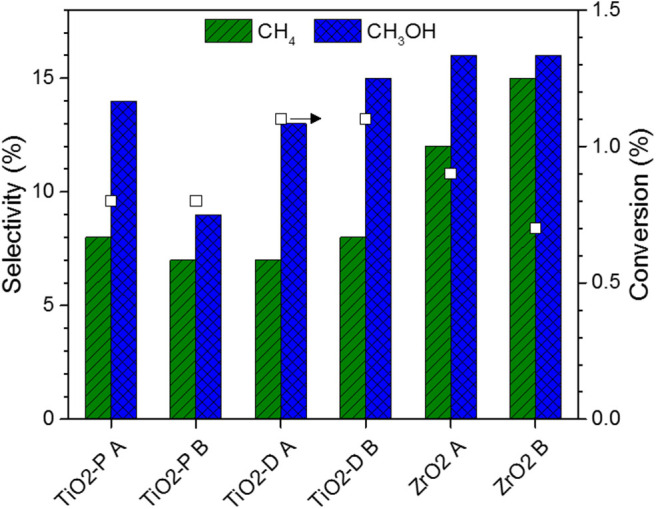
Conversion and selectivity for supported Mo nitride catalysts. Catalytic conditions: 400 mg of catalyst, 50 ml min^−1^, 250°C, 30 bar, H_2_:CO_2_ = 3:1, 150 min on stream after stabilization of the temperature.

The series of Mo nitride catalysts supported on TiO_2_-P, TiO_2_-D, and ZrO_2_ was tested for the hydrogenation of CO_2_ in the same conditions as those used for the carbides. The performances at quasi-steady state are reported in Figure 8. The conversions are in the range 0.7–1.1%, which implies that the nitride catalysts are less active than their carbide counterparts. Similarly to the carbides, the activity of supported MoN varies in the order: TiO_2_-D > P25 TiO_2_-P ≈ ZrO_2_.

The selectivity to CO, methane, and methanol are in the ranges 69–84, 7–15, and 9–16%, respectively. The highest CO selectivity are those of the TiO_2_-supported samples, while the highest methane and methanol selectivity are those of ZrO_2_-supported catalysts. With respect to the carbides, the CO/CH_4_ selectivity ratio is higher for the nitrides. Moreover, no ethane is produced on these catalysts, which suggests a weaker adsorption of CO_2_, leading to the absence of CO_2_ decomposition to atomic carbon (Xu et al., 2014).

Finally, despite the different degrees of nitridation provided by methods A and B, the synthesis method poorly affects the catalytic performance, confirming the low activity of N-terminated sites. Moreover, Mo_2_N catalysts are characterized by the presence of Mo^δ+^ (2 ≤ δ^+^ < 4) (Perret et al., 2012), which confirms that the oxidation states of Mo affect the hydrogenation of CO_2_.

γ-Mo_2_N synthesized using reduction–nitridation under NH_3_ at 700°C was reported to be active and selective for the RWGS reaction (Yao et al., 2019). At 350°C, ca. 2.5% conversion and a CO selectivity above 98% was measured. Our results confirm that Mo_2_N is able to uptake and activate CO_2_ as ca. 1% conversion was observed under our reaction conditions. However, supported molybdenum carbides outperform the nitride counterparts. Simple ways of increasing CO_2_ conversions consist in decreasing the reactant gas flow rate and increasing catalyst weight, as done during the preliminary tests (Table S8).

## Conclusion

Molybdenum carbide and nitride nanoparticles supported on TiO_2_ and ZrO_2_ were evaluated in the hydrogenation of CO_2_ for the first time. The catalysts were synthesized by impregnation followed by reduction–carburization or reduction–nitridation. Synthesis parameters such as temperature, nature, and composition of the gas stream, and space velocity were varied. Characterization by TEM/STEM and XRD showed the formation of cubic MoC and Mo_2_N nanoparticles. Different degrees of carburization (Mo/C molar ratio) and nitridation (Mo/N) were obtained depending on the synthesis conditions.

For MoC/TiO_2_, employing a low concentration of hydrocarbon in hydrogen and a low temperature during the synthesis results in a lower C/Mo ratio and a higher activity. Moreover, the presence of graphite inhibits the reaction. The carbide catalysts generate mainly CO, along with methane, ethane, and methanol. The nature of the support affects the catalytic performances, which are higher for TiO_2_-D (commercial DT51) support. The preparation conditions and the support affect the selectivity to methanol, which varies from 1 to 11%. The Mo nitride catalysts are less active than their carbide counterparts. The Mo/N molar ratio does not affect the conversion and the product distribution. Regarding the carbide catalysts, the presence of carbon vacancies seems to be crucial. Further work will focus on the control of the amount of these vacancies in an attempt to increase the catalytic activity and tune the selectivity.

## Data Availability Statement

The original contributions presented in the study are included in the article/Supplementary Materials, further inquiries can be directed to the corresponding author/s.

## Author Contributions

MA and AN conducted the experiments. MA wrote the manuscript. RC set up the catalytic bench and helped with operating the experiments. MJ and CP assisted in supervising the work, data analyzing, and quality controlling. LP and NP designed the study, acquired the founding, supervised the work, and reviewed the manuscript.

## Conflict of Interest

The authors declare that the research was conducted in the absence of any commercial or financial relationships that could be construed as a potential conflict of interest.
