# Supplementary material for: Supported Molybdenum Carbide and Nitride Catalysts for Carbon Dioxide Hydrogenation
Source: Front Chem. 2020 Jun 9;8:452. doi: 10.3389/fchem.2020.00452 (PMC7296157; doi:10.3389/fchem.2020.00452)
Supplement: Supplementary file 1 [file Data_Sheet_1.PDF]

Supported molybdenum carbide and nitride catalysts for carbon dioxide hydrogenation

Marwa Abou Hamdan, Abdallah Nassereddine, Ruben Checa, Mohamad Jahjah, Catherine Pinel, Laurent Piccolo, Noémie Perret

Supplementary material

Table S1. List of the supported molybdenum carbide catalysts synthesised with CH_4/H_2 (GHSV = 1090 h^{-1}) with the corresponding preparation conditions, C/Mo atomic ratio derived from elemental analyses and support composition (% phase). The name of the samples (MoC) does not reflect their C/Mo ratio.

Catalyst name	Gas stream	T_{max} (°C)	C/Mo	% phase[a]
MoC_{5M-700}/TiO_2-P	5% CH_4/H_2	700	0.3	50
$MoC_{10M-700}/TiO_2$-P	10% CH_4/H_2	700	0.7	67
$MoC_{20M-700}/TiO_2$-P	20% CH_4/H_2	700	0.5	72
$MoC_{20M-600}/TiO_2$-P	20% CH_4/H_2	600	0.2	75
$MoC_{20M-800}/TiO_2$-P	20% CH_4/H_2	800	0.5	n.a.
$MoC_{20M-700}/TiO_2$-D	20% CH_4/H_2	700	0.7	100

[a] anatase/rutile composition (% anatase)

Table S2. Mo and C content, crystallite size (d_{MoC}) and lattice parameters (a) of MoC, crystallite size ($d_{anatase}$, d_{rutile}, $d_{monoclinic}$, $d_{tetragonal}$) and surface areas (SA), for the different supported molybdenum carbide catalysts.

**Catalysts**	**Mo (wt%)[a]**	**C (wt%)[b]**	**d_{MoC} (nm)**	**a (Å)**	**$d_{anatase}$ (nm)**	**d_{rutile} (nm)**	**SA (m^2 g^{-1})**
MoC/TiO_2-P	11.8	2.2	2	4.248	26	52	45
MoC_{5E-700}/TiO_2-P	9.6	0.8	3	4.234	27	63	43
$MoC_{10E-700}$/TiO_2-P	9.6	0.8	3	4.246	27	66	47
$MoC_{20E-700}$/TiO_2-P	9.4	1.2	3	4.270	29	11	49
$MoC_{20E-600}$/TiO_2-P	9.7	0.8	3	n.a.	28	53	54
$MoC_{20E-800}$/TiO_2-P	8.9	1.4	n.a.	n.a.	n.a.	n.a.	51
MoC_{5M-700}/TiO_2-P	9.7	0.4	2	4.224	28	82	n.a.
$MoC_{10M-700}$/TiO_2-P	9.3	0.8	3	4.227	27	71	47
$MoC_{20M-700}$/TiO_2-P	9.5	0.6	3	4.256	27	62	50
$MoC_{20M-600}$/TiO_2-P	9.7	0.3	n.a.	n.a.	27	45	54
$MoC_{20M-800}$/TiO_2-P	10.0	0.6	3	n.a.	n.a.	n.a.	28
$MoC_{20E-700}$/TiO_2-D	9.1	1.6	2	4.251	24	-	84
$MoC_{20M-700}$/TiO_2-D	9.5	0.8	2	4.237	26	-	n.a.
**Catalysts**	**Mo (wt%)[a]**	**C (wt%)[b]**	**d_{MoC} (nm)**	**a (Å)**	**$d_{monoclinic}$ (nm)**	**$d_{tetragonal}$ (nm)**	**SA (m^2 g^{-1})**
$MoC_{20E-700}$/ZrO_2	9.2	2.1	2	4.187	11	6	129

[a] Weight percentage, analysed by ICP; [b] weight percentage, analysed by carbon analysis; n.a. not available

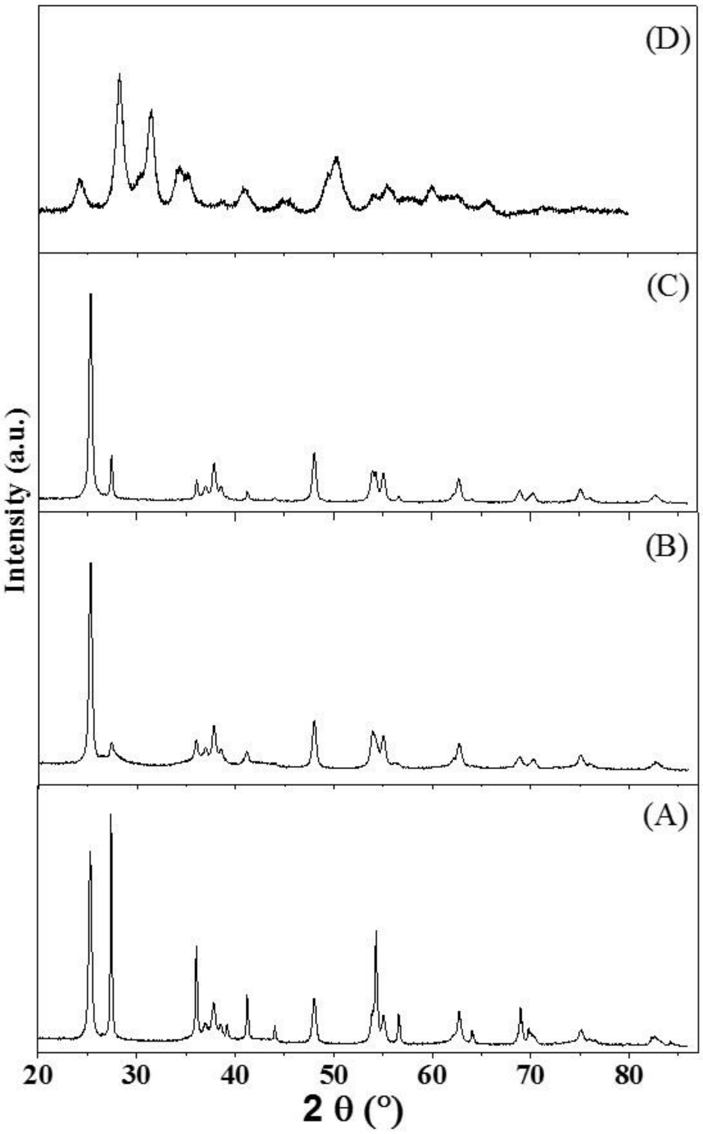

Figure S1. XRD diffraction patterns of the catalysts (A) MoC_{5E-700}/TiO_2-P, (B) $MoC_{20E-700}/TiO_2$-P, (C) $MoC_{20E-600}/TiO_2$-P, (D) $MoC_{20E-700}/ZrO_2$.

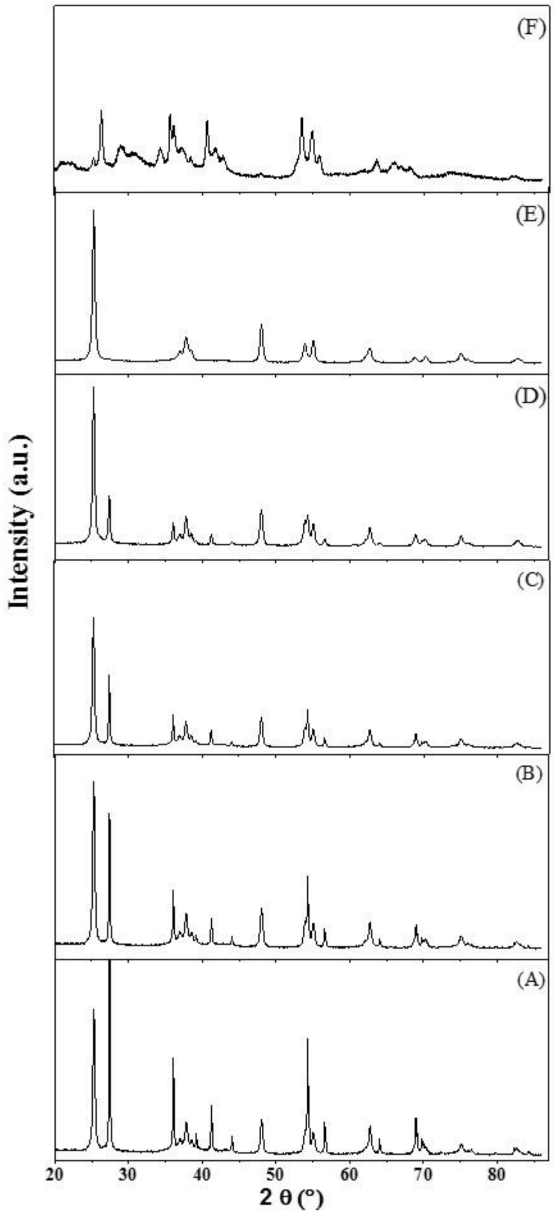

Figure S2. XRD diffraction patterns of the catalysts (A) MoC_{5M-700}/TiO_2-P, (B) $MoC_{10M-700}/TiO_2$-P, (C) $MoC_{20M-700}/TiO_2$-P, (D) $MoC_{20M-600}/TiO_2$-P, (E) $MoC_{20M-700}/TiO_2$-D and (F) $MoC_{20M-800}/TiO_2$-P.

Figure S3. TEM images and electron diffraction pattern (corresponding to the red circle in the image) of $MoC_{20E-700}/TiO_2$-D.

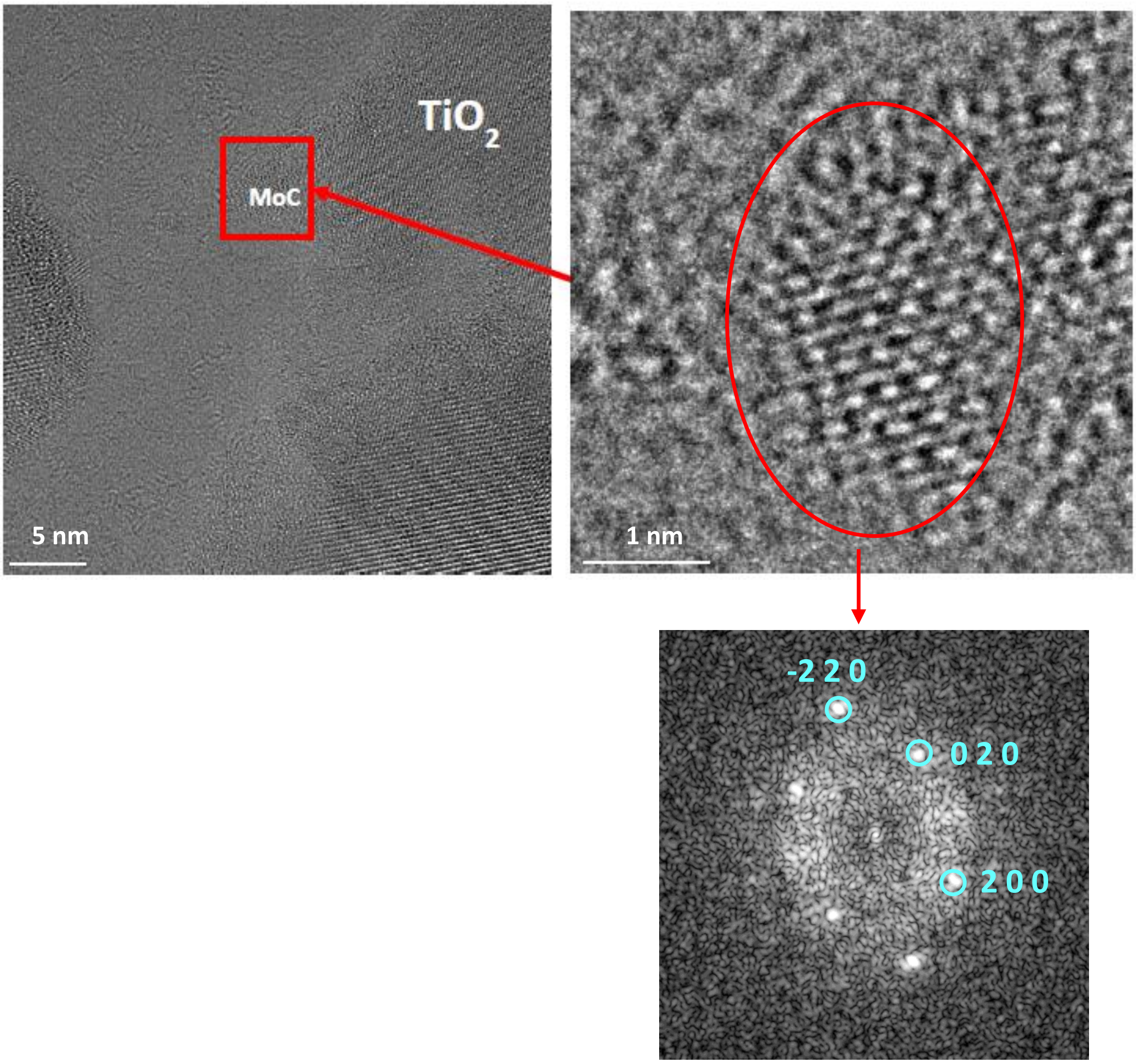

Table S3. Comparison of d-spacing and angles obtained from TEM analysis of $MoC_{20E-700}/TiO_2$-D (Figure S3) with theoretical values corresponding to cubic fcc MoC, code ICSD 197178.

**hkl**	**d_{exp} (nm)**	**d_{theo} (nm)**	**$angle_{exp}$ (°)**	**$angle_{theo}$ (°)**
200	0.2146	0.2141	0.00	0.00
020	0.2199	0.2141	89.47	90.00
-220	0.1522	0.1514	13513	135.00

Figure S4. TEM image and electron diffraction pattern (corresponding to the red square in the image) of $MoC_{10E-700}/TiO_2$-P.

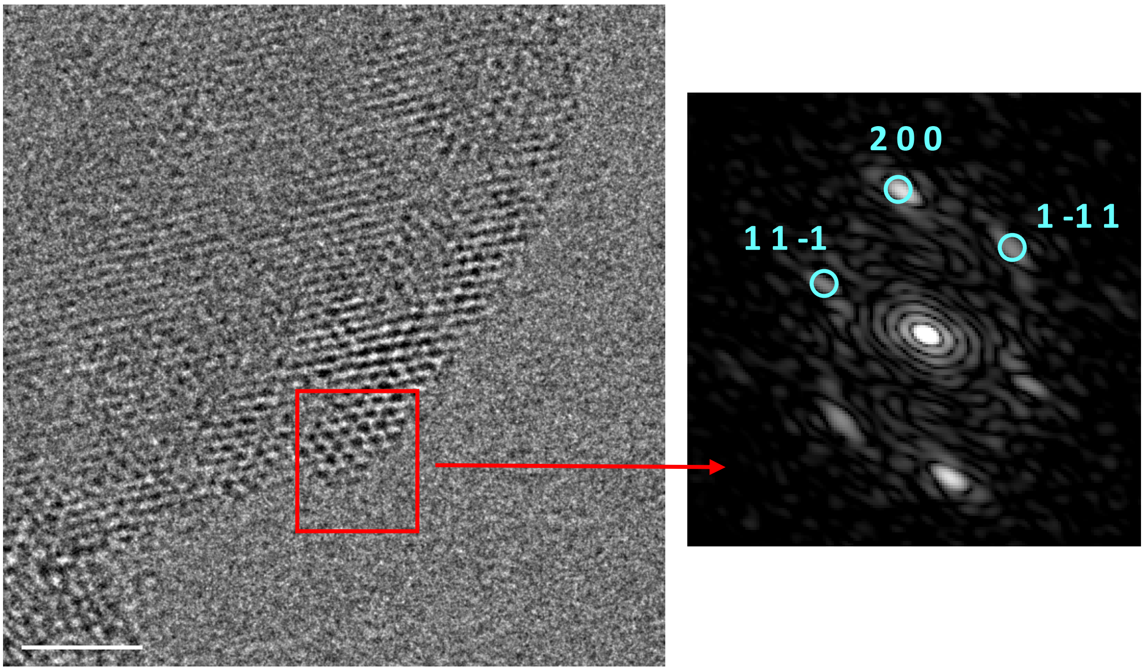

Table S4. Comparison of d-spacing and angles obtained from TEM analysis of $MoC_{10E-700}/TiO_2$-P (Figure S4) with theoretical values corresponding to cubic fcc MoC, code ICSD 197178.

**hkl**	**d_{exp} (nm)**	**d_{theo} (nm)**	**$angle_{exp}$ (°)**	**$angle_{theo}$ (°)**
11-1	0.2521	0.2472	0.00	0.00
200	0.2070	0.2141	55.52	54.74
1-11	0.2652	0.2472	109.54	109.47

Table S5. Mo and C content, crystallite size (d_{MoN}) of MoN, anatase/rutile composition (% anatase), monoclinic/tetragonal composition (% monoclinic) and crystallite size ($d_{anatase}$, d_{rutile}, $d_{monoclinic}$, $d_{tetragonal}$), for the different supported molybdenum carbide catalysts.

**Catalysts**	**supports**	**method**	**Mo (wt%)[a]**	**N (wt%)[b]**	**d_{MoN} (nm)**	**$d_{anatase}$ (nm)**	**d_{rutile} (nm)**
MoN/TiO_2-P	TiO_2 P25	A	9.1	0.6	3	28	51
MoN/TiO_2-P	TiO_2 P25	B	9.0	0.7	3	28	48
MoN/TiO_2-D	TiO_2 DT51	A	9.3	0.8	2	26	-
MoN/TiO_2-D	TiO_2 DT51	B	9.3	0.9	2	26	-
**Catalysts**	**supports**	**method**	**Mo (wt%)[a]**	**N (wt%)[b]**	**d_{MoN} (nm)**	**$d_{monoclinic}$ (nm)**	**$d_{tetragonal}$ (nm)**
MoN/ZrO_2	ZrO_2	A	9.3	0.6	2	11	6
MoN/ZrO_2	ZrO_2	B	9.3	0.7	2	11	6

[a] Weight percentage, analysed by ICP; [b] weight percentage, analysed by nitrogen analysis

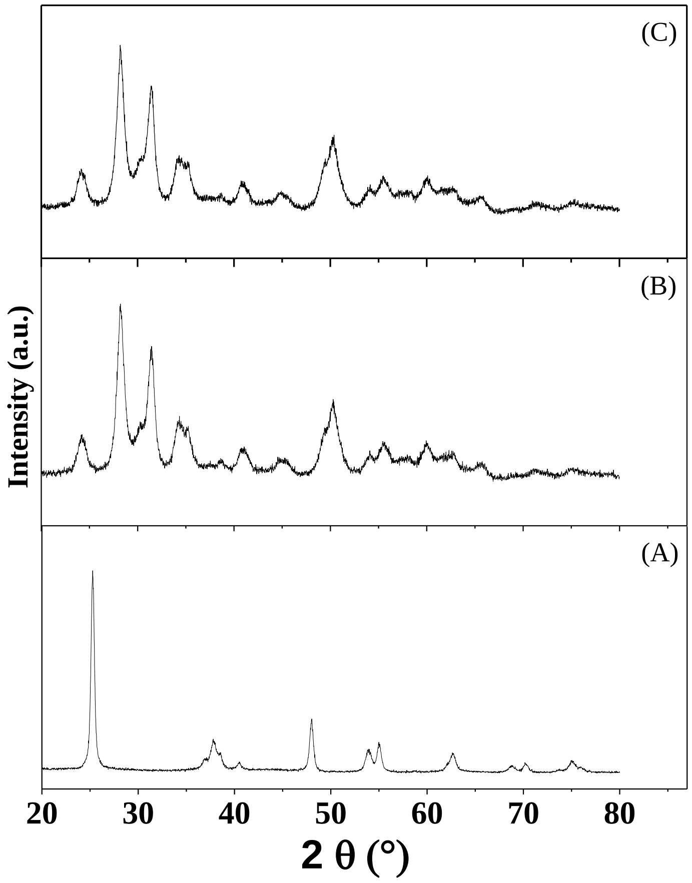

Figure S5. XRD diffraction patterns of the catalysts (A) MoN_A/TiO_2-D, (B) MoN_A/ZrO_2, (C) MoN_B/ZrO_2.

Figure S6. TEM image and electron diffraction pattern (corresponding to the red square in the image) of MoN_B/TiO_2-D.

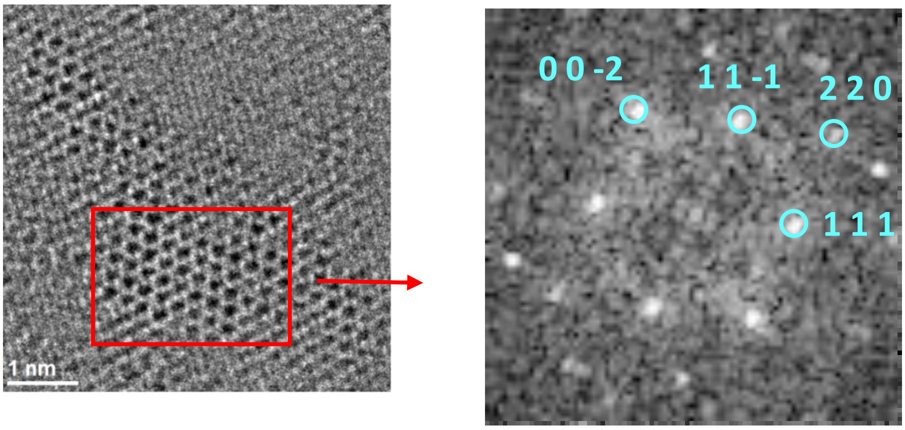

Table S6. Comparison of d-spacing and angles obtained from TEM analysis of MoN_B/TiO_2-D (Figure 4B and Figure S6) with theoretical values corresponding to cubic Mo_2N, code ICSD 251625.

hkl	d_{exp} (nm)	d_{theo} (nm)	$angle_{exp}$ (°)	$angle_{theo}$ (°)
1 1 1	0.2412	0.2420	0.00	0.00
2 2 0	0.1499	0.1482	35.89	35.26
1 1 -1	0.2371	0.2420	71.38	70.53
0 0 -2	0.2074	0.2096	126.09	125.26

Figure S7. TEM image and electron diffraction pattern (corresponding to the red square in the image) of MoN_A/ZrO_2.

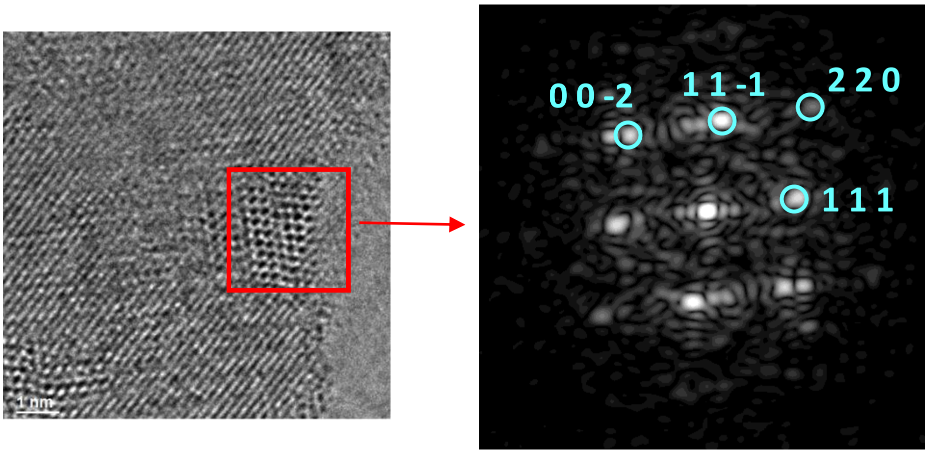

Table S7. Comparison of d-spacing and angles obtained from TEM analysis of MoN_A/ZrO_2 (Figure 4C and Figure S7) with theoretical values corresponding to cubic Mo_2N, code ICSD 251366.

hkl	d_{exp} (nm)	d_{theo} (nm)	$angle_{exp}$ (°)	$angle_{theo}$ (°)
1 1 1	0.2415	0.2404	0.00	0.00
2 2 0	0.1489	0.1472	36.10	35.26
1 1 -1	0.2399	0.2404	72.6	70.53
0 0 -2	0.2033	0.2081	126.73	125.26

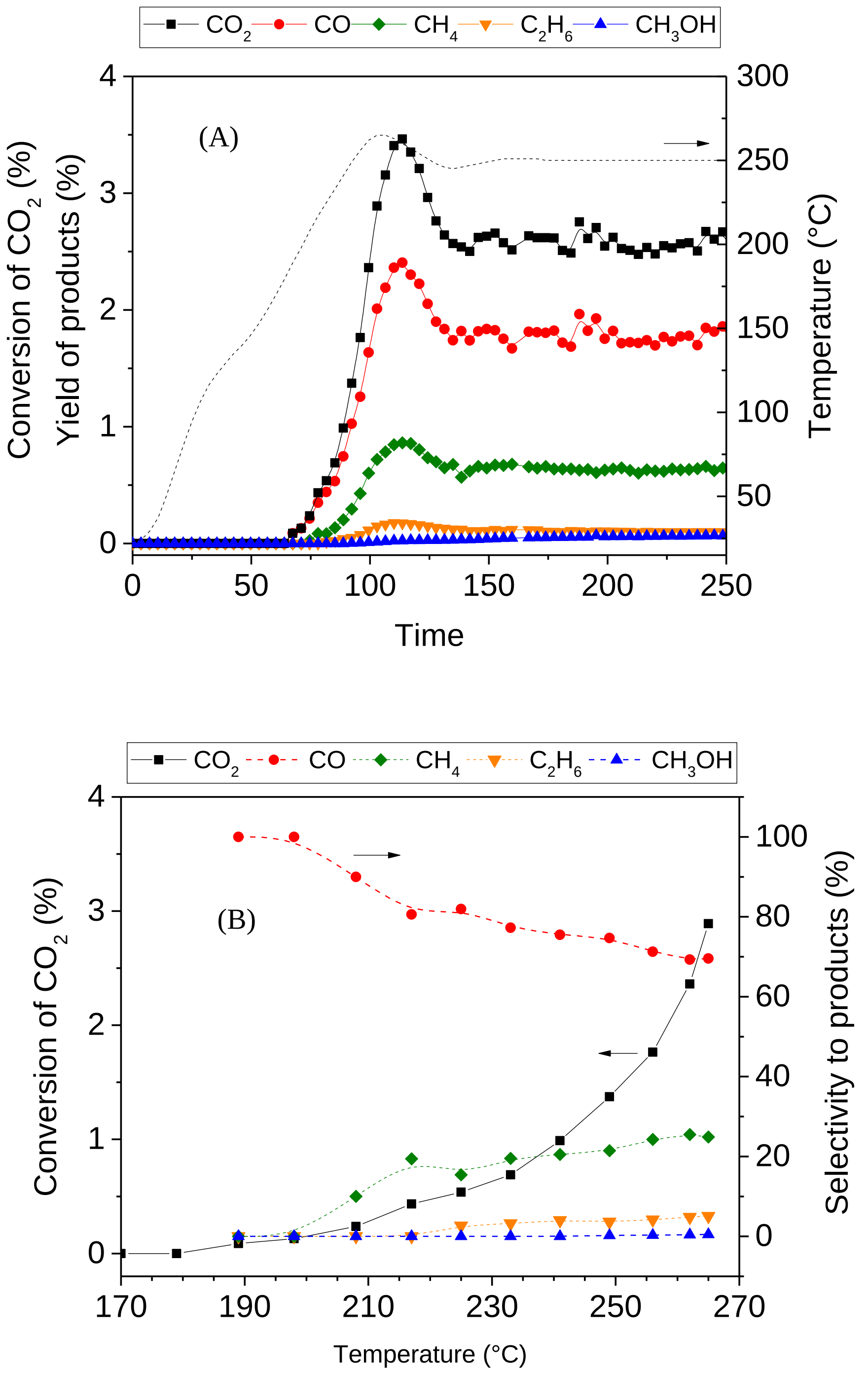

Figure S8. (A) evolution of CO_2 conversion and products yields in function of time **(B)** evolution of CO_2 conversion and products selectivity in function of temperature, during the hydrogenation of CO_2 over 400 mg of MoC/TiO_2-P at 250 °C and 20 bar total pressure, with a total flow rate of 30 mL min^{-1} of H_2 /CO_2/N_2 with a H_2:CO_2 ratio of 5:1. *Note:* the dashed line in (A) corresponds to the temperature.

Table S8. Reaction conditions and associated catalytic results for MoC/TiO_2-P after 280 min on stream.

Reaction conditions						Catalytic performances				
**Entry**	**Catalyst mass (mg)**	**flow (mL min^{-1})**	**T (°C)**	**P (bar)**	**H_2: CO_2 ratio**	**CO_2 conversion (%)**	**Products selectivity (%)**			
							**CO**	**CH_4**	**C_2H_6**	**CH_3OH**
R1	400	30	250	20	5:1	2.5	69	25	3	3
R2	400	50	250	30	3:1	2.0	71	23	3	3
R3	400	50	250	30	3:1	2.0	72	22	3	3
R4	800	50	250	30	3:1	4.0	70	25	4	1
R5	800	30	250	30	3:1	8.0	72	24	4	< 1
R6	800	10	250	30	3:1	12.0	61	33	6	< 1
R7	800	50	300	30	3:1	11.4	79	18	3	< 1
R8	800	50	200	30	3:1	0.5	72	26	0	2
R9	400	50	250	30	5:1	3.0	67	26	4	3
R10[a]	800	50	250	30	3:1	7.2	73	23	3	1

[a] This catalyst was not passivated after synthesis.

<u>Comments associated with Table S8:</u>

- Prior to the tests of CO_2 hydrogenation over molybdenum carbide catalysts supported on titanium oxide, two blank tests were performed: one with empty reactor, and the second with TiO_2-P support. In both cases no conversion was observed (< 0.1%).
- Entry R1 correspond to the results of **Figure 5**.
- The reproducibility was checked by conducting two reactions under the same conditions using two batches of MoC/TiO_2 synthesized in the same way (R2 and R3). The results were equivalent within ± 5% in terms of CO_2 conversion (2.0%) and products selectivity.
- When the catalyst weight was doubled (R4), i.e. the weight hourly space velocity (WHSV) was divided by two, the conversion increased by a factor of 2, which indicates the absence of mass transfer limitations. Moreover, the products distributions were fairly constant.
- When increasing the H_2:CO_2 molar ratio from 3:1 (R2) to 5:1 (R9), the CO_2 conversion increases slightly from 2.0% to 3.0% without modification of the selectivity.
- The non-passivated catalyst, R10, is to be compared with the passivated one R4.
- The total flow rate of the reactant mixture was decreased from 50 mL min^{-1} (R4) to 30 mL min^{-1} (R5), and then to 10 mL min^{-1} (R6). As expected, decreasing the flow, i.e. WHSV, increased the conversion to the same extent (from 4% to 12%). However, with the low flow rates the kinetic was not stabilized after 280 min on stream (**Figure below**).

Figure associated with Table S8: Evolution of CO_2 conversion and selectivities during the hydrogenation of CO_2 over 800 mg of MoC/TiO_2-P at 250 °C and 30 bar, with 10 mL/min flow of H_2 / CO_2/ N_2 with H_2:CO_2 ratio 3:1.

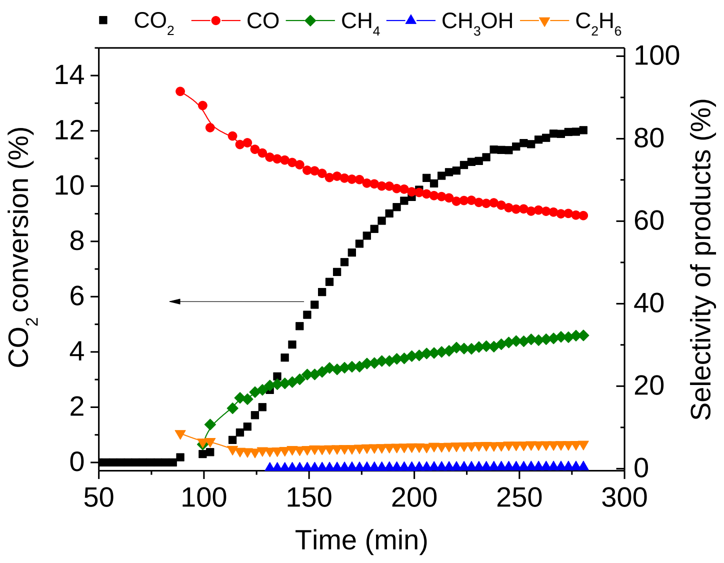

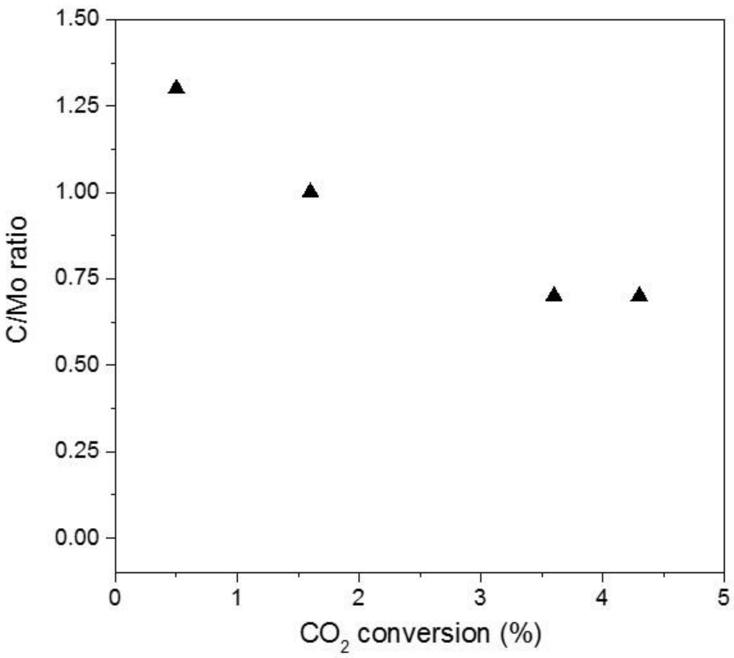

Figure S9. C/Mo ratio as a function of CO_2 conversion over MoC/TiO_2-P, independently of the temperature and amount of ethane used for carburization. Catalytic conditions: 400 mg of catalyst, 50 mL min^{-1}, 250 °C, 30 bar, H_2:CO_2 = 3:1.

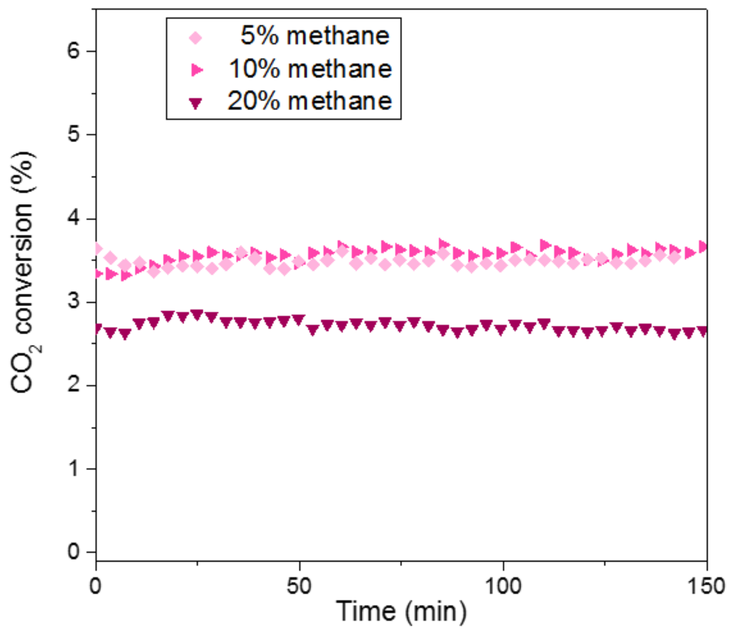

(A)

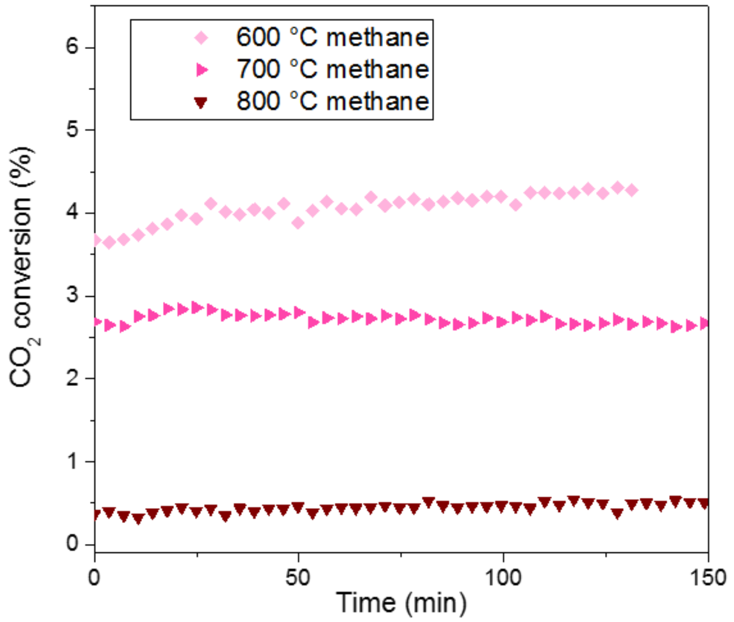

(B)

Figure S10. CO_2 conversion over MoC/TiO_2-P as a function of the amount of methane used for carburization at 700 °C (A) and the carburization temperature used for 20% methane (B). Catalytic conditions: 400 mg of catalyst, 50 mL min^{-1}, 250 °C, 30 bar, H_2:CO_2 = 3:1. 150 min on stream after stabilization of the temperature.

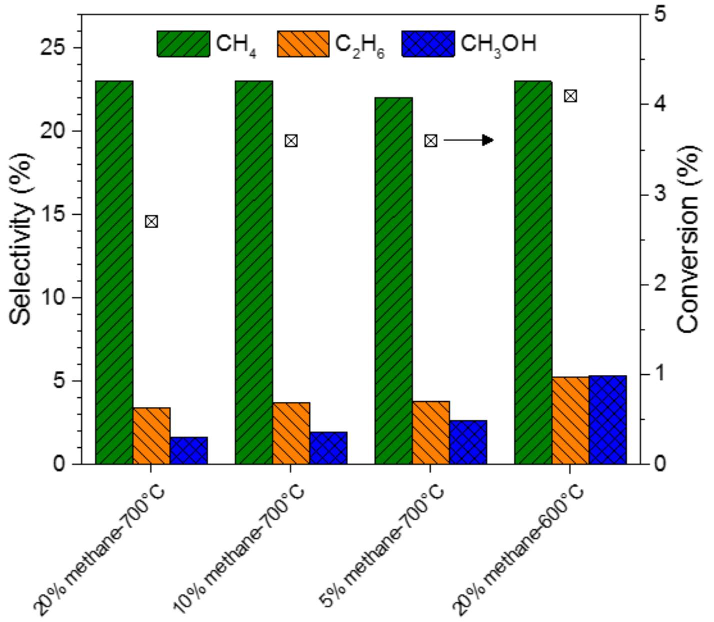

Figure S11. Selectivity to methane, ethane, and methanol, and conversion of MoC/TiO_2-P as a function of the carburizing methane concentration (5%, 10%, or 20% in H_2), and the carburization temperature (600 or 700 °C). Catalytic conditions: 400 mg of catalyst, 50 mL/min, 250 °C, 30 bar, H_2:CO_2 = 3:1, 150 min on stream after stabilization of the temperature.

Table S9. Effect of the support on the catalytic performances.

Catalyst	CO_2 conversion (%)	Products selectivity (%)[a]			
		CO	CH_4	C_2H_6	CH_3OH
$MoC_{20M\text{-}700}/TiO_2$-P	2.7	72	23	3	2
$MoC_{20M\text{-}700}/TiO_2$-D	3.5	63	20	7	10

[a] Reaction conditions: 400 mg of catalyst, 50 mL/min, 250° C, 30 bar, 3:1 H_2:CO_2 ratio, 150 min on stream after stabilization of the temperature.

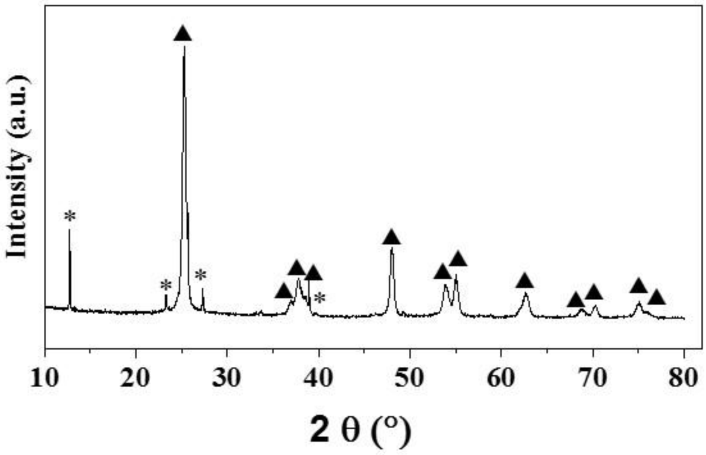

Figure S12. XRD diffraction pattern of MoO_3/TiO_2-D; anatase (▲) and orthorhombic MoO_3 (∗).
